# Effects of cycle duration of an external electrostatic field on anammox biomass activity

**DOI:** 10.1038/srep19568

**Published:** 2016-01-22

**Authors:** Xin Yin, Sen Qiao, Jiti Zhou

**Affiliations:** 1 Key Laboratory of Industrial Ecology and Environmental Engineering (Ministry of Education, China), School of Environmental Science and Technology, Dalian University of Technology, Dalian 116024, P.R. China; 2 Jiangxi Provincial Key Laboratory of Water Resources and Environment of Poyang Lake, Jiangxi Institute of Water Sciences, Nanchang 330029, P.R. China

## Abstract

In this study, the effects of different cycle durations of an external electrostatic field on an anammox biomass were investigated. The total application time per day was 12 h at 2 V/cm for different cycle durations (i.e., continuous application-resting time) of 3 h-3 h, 6 h-6 h, and 12 h-12 h. Compared with the control reactor, the nitrogen removal rates (NRRs) increased by 18.7%, 27.4% and 8.50% using an external electrostatic field application with a continuous application time of 3 h, 6 h and 12 h. Moreover, after the reactor was running smoothly for approximately 215 days under the optimal electrostatic field condition (mode 2, continuous application-rest time: 6 h-6 h), the total nitrogen (TN) removal rate reached a peak value of approximately 6468 g-N/m^3^/d, which was 44.7% higher than the control. The increase in 16S rRNA gene copy numbers, heme *c* content and enzyme activities were demonstrated to be the main reasons for enhancement of the NRR of the anammox process. Additionally, transmission electron microscope observations proved that a morphological change in the anammox biomass occurred under an electrostatic field application.

Anaerobic ammonium oxidation (anammox) has already been recognized as an innovative nitrogen removal technology for wastewater treatment^1^,^2^. Compared with the conventional biological processes (nitrification-denitrification), the anammox process offers significant advantages, such as no demand for oxygen and organic carbon, low sludge production and reduced CO_2_ or N_2_O emissions^3^. In 2010, Tang *et al*. reported a very high nitrogen removal rate of 74.3–76.7 kg-N/m^3^/d in a lab-scale anammox UASB reactor, which demonstrated the high potential of the anammox process for biological nitrogen removal from wastewater^4^. However, such a high nitrogen removal rate (NRR) was achieved by continuously adding anammox biomass into the targeted reactor, so the biomass concentration increased up to 42.0–57.7 g-VSS/L. The low growth rate in this condition still poses difficult technological challenges even though most studies reported encouraging results for the application of anammox^5^,^6^,^7^. The start-up of the first full-scale anammox reactor took almost 3.5 years^8^. Consequently, enhancing the activity of the anammox bacteria or shortening the start-up period of anammox reactors is a subject of great interest and challenge.

Recently, several exciting studies have utilized external field energy, such as a magnetic field and low intensity ultrasound, to increase the activity of anammox bacteria^9^,^10^. In fact, an external electrostatic field might be another effective approach to enhance anammox biomass activity. The concept of applying electrostatic fields to cells to influence cell biology has been used in biological research for several decades. The specific sensitivity of biological cells towards electrostatic fields has been used for various purposes, such as cell growth, cell death, diagnostics, sensing devices, healing or gene transfer purposes^11^,^12^,^13^. When cells are exposed to electrostatic fields, polarization of the cell membrane and its components occurs, which may lead to the following phenomena, rotation, cell membrane permeability and osmotic imbalance^14^. Thus, it is possible that mechanical instability of the membranes of anammox cells could be created when an electrical field is applied that causes a critical membrane potential to induce tension to increase the cell membrane permeability. Moreover, some researchers have shown that a pulsed electrostatic field could promote the activity of some enzymes, especially for enzymes that have heme^15^. Additionally, it is reasonable to assume that the application of a low level electrostatic field might promote mass transfer of the anammox cells because of a change in the membrane morphology. Furthermore, the electrostatic field may enhance the activities of the key enzymes by the conformational change and faster transfer of heme. However, until now, few studies that focus on the effects of applying an electrostatic field on the activity of anammox biomass and key enzymes activities existed.

Our preliminary experimental results demonstrated that the anammox biomass activity could be increased using an external electrostatic field (2 V/cm), while 24 h of continuous application would definitely depress the anammox biomass activity when in the range of 1 and 4 V/cm^16^. In addition to the electrostatic field, application time is predicted to be another key factor that influences anammox biomass activity intensity. Thus, the main aim of this study was to investigate the effects of a low level external electrostatic field on the activity of anammox biomass with different application times. Furthermore, variations in the heme *c* contents, enzymes activities, 16S rRNA gene numbers of anammox bacteria and cell morphology variation were explored.

## Results and Discussion

### Continuous Experiment

**Figure 1:**
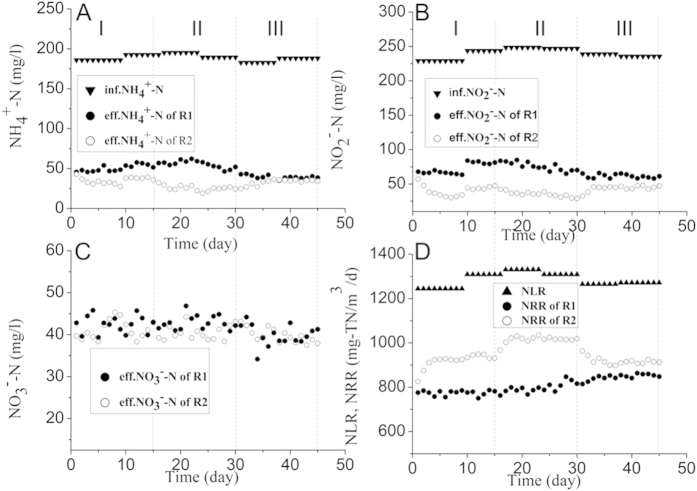
Comparison of nitrogen removal performance of two reactors in phases I-III. (**A**) NH_4_^+^-N; (**B**) NO_2_^−^-N; C, NO_3_^−^-N; D, NLR and NRR.

Figure 1 presented the relationship between the application modes and corresponding anammox activities. There was an observable increase in the nitrogen removal performance with an applied electrostatic field compared with the control experiments. The enhancement of biological activity changed with the continuous application time of the electrostatic field. At the end of phase I (mode 1, continuous application-rest time: 3 h-3 h), the TN removal efficiency of R2 with an electrostatic field applied was 71%, which was approximately 18.3% higher than the control reactor (R1). Subsequently, the nitrogen removal efficiency continued to increase after the continuous application time increased to 6 h (mode 2, continuous application-rest time: 6 h-6 h). On day 30 of the run, the TN removal efficiency of R2 climbed to 78%, while the efficiency of R1 was quite stable at approximately 62%. In contrast, when the continuous application time was greater than 6 h in one cycle, the activity of the anammox biomass did not further increase but rather decreased. During phase III, the TN removal efficiency of R2 declined to 72% after the continuous application time increased to 12 h in one cycle (mode 3, continuous application-rest time: 12 h-12 h). These continuous experimental results demonstrated that the cycle duration of an external electrostatic field played a distinct and key role on the activity of the anammox biomass. The peak positive effect of the electrostatic field was application mode 2 with a cycle duration of 6 h. Thus, this mode (mode 2, continuous application-resting time: 6 h-6 h,) was utilized for the following continuous experiments (phase IV) to examine its long-term effects on the activity of the anammox biomass.

**Figure 2:**
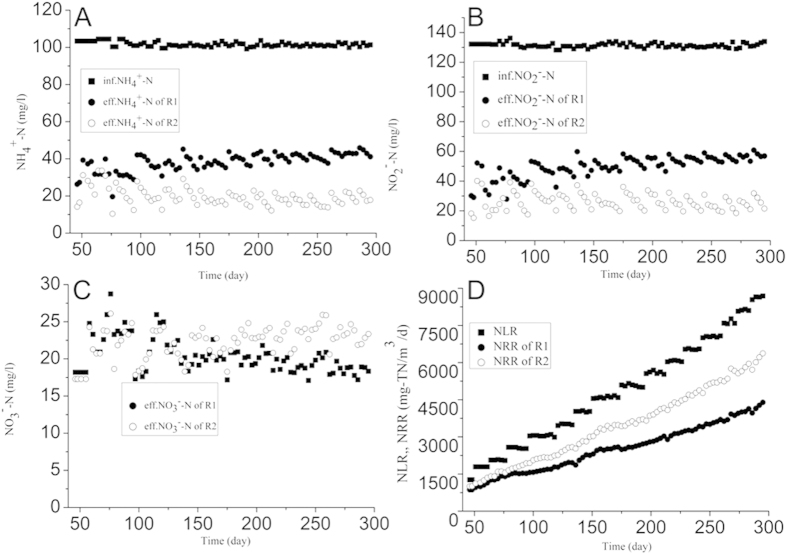
Comparison of nitrogen removal performance of two reactors in phase IV. (**A**) NH_4_^+^-N; (**B**) NO_2_^−^-N; (**C**) NO_3_^−^-N; (**D**) NLR and NRR.

In phase IV, a short hydraulic retention time (HRT) was applied as the main method to increase the NLRs of both reactors with constant influent substrates concentrations. As shown in Fig. 2, the NRRs of both reactors were 867 and 1002 g-N/m^3^/d on day 46. The inhibition of the anammox biomass in R2 because of the mal-effects of the external electrostatic field during phase III resulted in the almost the same nitrogen removal performance for both reactors. In phase IV, the NRR of R2 rapidly increased and then remained constant with better stable nitrogen removal performance than R1. For instance, the NRR of R2 began to increase only 9 days after the application mode returned to mode 2 (mode 2, application-rest time: 6 h-6 h), which was approximately 16.7% higher than R1 on day 55. During the rest of the running days, the nitrogen removal performance was always higher than R1. At the end of phase IV, the NLR of the two reactors increased to 8641 g-N/m^3^/d, while the NRRs of both reactors reached 4470 and 6468 g-N/m^3^/d. In our study, these two reactors were operated under the same conditions except whether the external electrostatic field was applied, but the nitrogen removal performance was very different between them. Hence, these results implied that an appropriate application of an external electrostatic field was the main reason for this difference in nitrogen removal performance.

So far, many types of field intensities, such as a low intensity ultrasound or magnetic field, have been used to increase the TN removal rate of the anammox process^9^,^10^. However, experimental results showed that the electrostatic field was a better approach (approximately a 44.7% increase) compared with the low intensity ultrasound (approximately a 25.5% increase) or magnetic field (approximately a 30% increase). Additionally, it was demonstrated that the cycle duration significantly impacted the beneficial effects of the electrostatic field. It was proven that the cycle duration of 6 h was the most optimal condition for application of an electrostatic field to the anammox process. Hence, an electrostatic field application with an appropriate application time would be a useful and reliable method to increase the nitrogen removal rate of an anammox reactor by enhancing the biological activity of the anammox biomass.

### Comparison of enzymes activities and qPCR results

Table 1 shows the variation of key enzymes activities of both reactors during the incubation time. Obviously, the activities of enzymes such as hydrazine dehydrogenase (HDH), nitrate reductase (NAR) and nitrite reductase (NIR) were greatly enhanced by the electrostatic field application for all three phases with different cycle durations. For instance, the HDH activity had a peak value of 1.84 μmol cytochrome *c* reduced/min/mg protein that was 1.36-fold higher than that of the control reactor within 45 days of operation at the end of phase II, which was obtained using mode 2 (continuous application-rest time: 6 h-6 h). Under the electrostatic field application of mode 3 (mode 3, continuous application-rest time: 12 h-12 h), the crude HDH activity dropped to 1.36 μmol cytochrome *c* reduced/min/mg protein at the end of phase III. In phase IV, the crude HDH activity of the two reactors was measured on days 80, 140, 200 and 260 and exhibited similar changes in both reactors. At the end of phase IV on day 260, the crude HDH activity of the anammox biomass in R2 increased to 2.92 cytochrome *c* reduced/min/mg protein. The peak value was approximately 3.1- and 1.6-fold higher than those of the seed sludge and R1, respectively. Obviously, the anammox bacteria with an applied electric field had different enzymes activities for different cycle durations.

**Figure 3:**
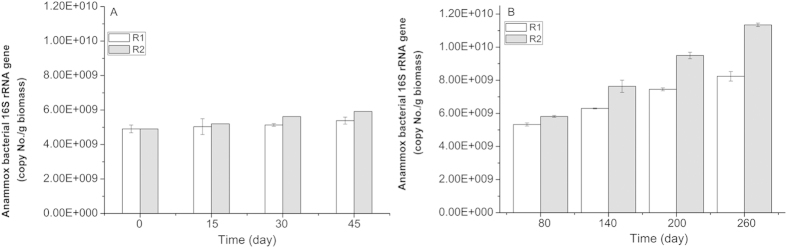
Comparison of 16S rRNA gene copy numbers of anammox bacteria in R1 and R2 during different phases. Each measurement was carried out in triplicate. (**A**) experimental data of phases I-III; (**B**) experimental data of phase IV. Data were shown as mean ± SD (n = 3). One-way ANOVA with Duncan’s multiple range test, *p < 0.05.

qPCR experiments can indirectly reflect the levels of anammox bacteria in both reactors. The variation of the 16S rRNA gene anammox bacterial copy numbers of both reactors was also investigated during the different phases (Fig. 3). As shown in Fig. 3A, the copy numbers in R1 had a stable trend upward within 45 days of operation (phase I-III). Although the same upward trend within 45 days of operation existed, the 16S rRNA gene anammox bacterial copy numbers in R2 showed little difference. After the continuous application time of the electrostatic field increased to 6 h (mode 2, application-rest time: 6 h-6 h), the value of R2 on day 30 (end of phase II) was approximately 8.3% more than on day 15, which was greater than 5.9% in phase I and 5.3% in phase III. This phenomenon indirectly demonstrated that the anammox bacteria growth rate of R2 in phase II was faster than in phase I and III. Moreover, after the reactor (R2) was running smoothly at the electrostatic field’s optimal condition, the 16S rRNA gene anammox bacterial copy numbers in R1 and R2 all demonstrated an increasing trend on running days 80–230. At day 260, the copy numbers in R2 reached 11.3 × 10^9^ copies/g biomass, which was approximately 40% higher than R1 (8.25 × 10^9^ copies/g biomass).

The variation of the 16S rRNA gene anammox bacterial copy numbers was ascribed to the following reasons. The electrostatic field of R2 with different cycle durations changed the enzymes activities, which increased the variation of the ammonium and nitrite consumption. To the best of our knowledge, the anammox biomass yield was proportional to the nitrogen consumption (0.066 ± 0.01 mol/mol ammonium)^17^. Under the electrostatic field condition, R2 always had relatively higher nitrogen removal efficiency during the incubation period corresponding to the long-term acceleration of the growth rate of the anammox biomass. Thus, the quantities of 16S rRNA gene copy numbers of the anammox cells in R2 with an electrostatic field application were more than R1 after long term cultivation.

### Comparison and observation of the cellular structure

**Figure 4:**
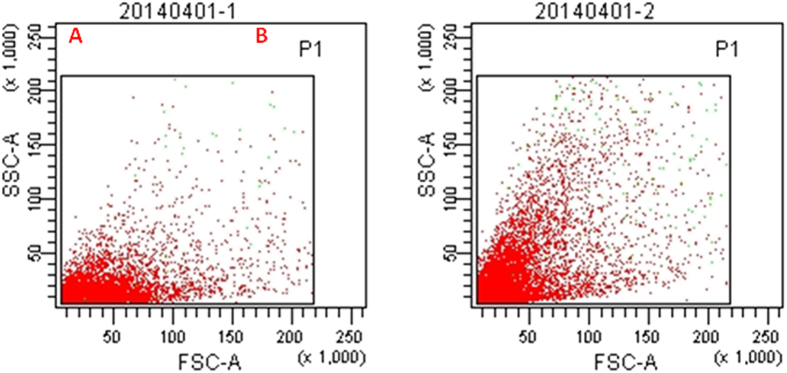
Anammox cells detected using PI dyes by flow cytometry. (**A**) was samples taken from R1 on day 200; (**B**) was samples taken from R2 on day 200.

To investigate the effects of the electrostatic field on the anammox cell structure, some sample cells were taken from the two reactors on day 200. The cells were first incubated with 30 mM propidium iodide (PI) for 15 min in the dark and then measured using flow cytometry. As shown in the flow cytometry images, green and red regions indicated intact cells and membrane-damaged cells, respectively (Fig. 4). Obviously, there were only a small little red region corresponding to minimal membrane-damaged cells in both R1 and R2, which confirmed the predication that a small intensity electrostatic field would not kill anammox cells but only create a morphological change in the anammox cells. Furthermore, although the forward scatters (FSC) intensities were similar in both reactors, the light intensity of the side scatter (SSC) in R2 was much stronger than in R1, as shown in Fig. 4. In general, the FSC intensity of the flow cytometry image reflected that the size of the cells and relevant information about the cell inner structures could be obtained from the measuring the SSC. Thus, some variations in the inner structure of anammox cells in R2 are expected because of the refractive index of the membrane and cytoplasm strengthening in an electrostatic field.

**Figure 5:**
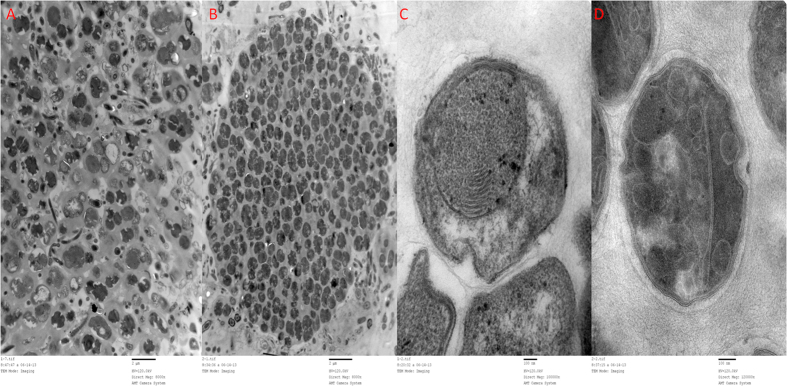
Comparison of the TEM observations of both reactors on day 200. (**A**,**C**) were samples taken from R1; (**B**,**D**) were samples taken from R2.

Samples of both reactors at day 200 were taken for TEM observations, as shown in Fig. 5. The distribution of bacterial flora of the two reactors showed evident differences. By comparing Figs. 5AB, it was obvious that the anammox bacteria were aggregated in R2 with the applied electrostatic field. Anammox cells in R2 displayed a compact cluster of in structure, while they were dispersed in R1 with a low cell-density. The increase in the 16S rRNA gene anammox bacterial copy numbers and higher cell-density further confirmed that the application of an electrostatic field with the appropriate condition had a significant positive effect on the growth rate of the anammox biomass. Moreover, abundant cells of R2 with an electrostatic field showed irregular shapes, especially forming many wrinkled areas at the anammoxosome edge. The final enzymes from the anammox reaction were localized exclusively in the matrix of the anammoxosome using immunogold localization^18^. The increase in the specific surface area of the anammoxosome membrane by the irregular shape and wrinkled areas might cause more enzymes to locate there. In addition, the more complex structure would result in a much stronger side scatter light, which is shown in the stronger SSC intensity in the flow cytometry images. Furthermore, with an electrostatic field application, many curvatures and compartments existed inside the anammoxosome, as shown in Fig. 5D. Jetten *et al*. assumed that the anammoxosome is used for energy metabolism by the highly folded anammoxosome membrane as the mitochondrial inner membrane (cristae)^19^. The additional compartments and membrane surface contributed to facilitating substrate turnover and storing large amounts of substrates^20^. The increase in the anammoxosome membrane surface area is assumed to provide more space to accommodate more substrates of the key enzymatic reaction (discussion later).

### Comparison of heme *c* contents and cytochrome *c*

**Figure 6:**
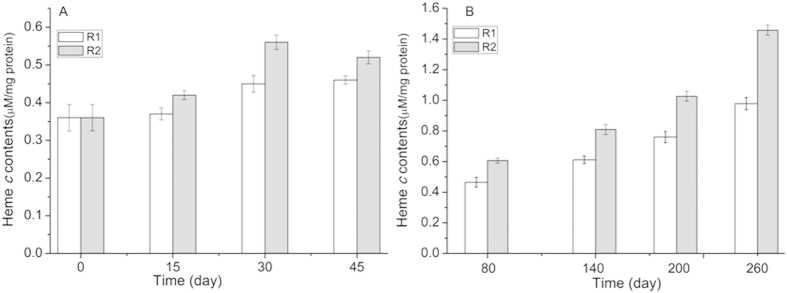
Comparison of heme *c* concentration of both reactors during different phases. Each measurement was carried out in triplicate. (**A**) experimental data of phase I-III; (**B**) experimental data of phase IV. Data were shown as mean ± SD (n = 3). One-way ANOVA with Duncan’s multiple range test, *p < 0.05.

Figure 6 depicted the comparison of heme *c* contents in both reactors at different phases. The heme *c* contents in R1 appeared to trend upward within the first 45 days of operation. The anammox biomass of R2 was approximately 0.56 μmol/mg protein on day 30, which was the highest heme *c* concentration at the end of phase II. During phase III, there was an obvious decrease in the heme *c* content in R2 with mode 3 (mode 3, application-rest time: 12 h-12 h). According to Fig. 6A, the variation of the heme *c* contents in R2 might be closely associated with the cycle durations of the electrostatic field. In phase IV, this value in R2 increased from 0.61 on day 80 to 1.45 μmol heme *c*/mg protein on day 260, which was approximately 49.4% higher than the value in R1. Heme *c* levels could indirectly reflect cytochrome *c* contents inside the anammox bacteria, because cytochrome *c* is involved in all of the key enzyme reactions of the anammox process^21^. The increase in heme *c* content predicted the increase in the cytochrome *c* levels inside the anammox bacteria, and further implied a possible increase in the activity of key enzymes.

**Figure 7:**
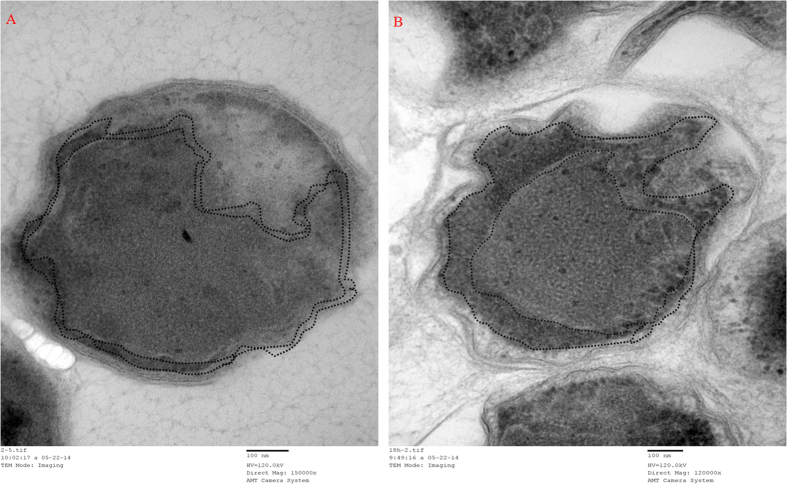
TEM observation showed the cytochrome peroxidase staining. (**A**) was samples taken from R1; (**B**) was samples taken from R2 under electrostatic field.

**Figure 8:**
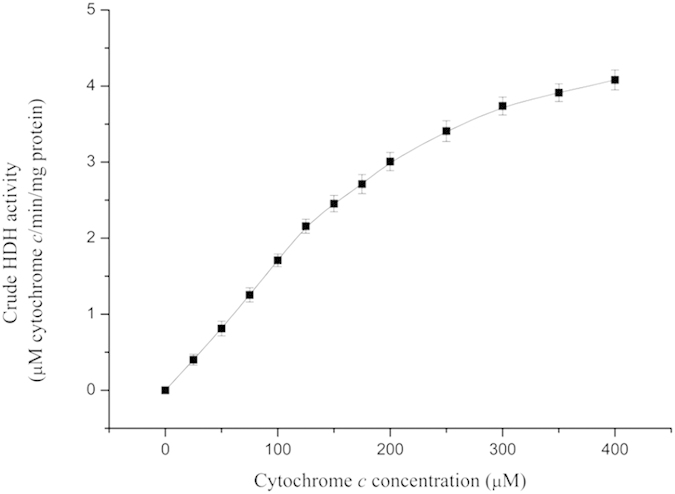
HDH activities with different concentration of cytochrome *c*. Each measurement was carried out in triplicate. Data were shown as mean ± SD (n = 3). One-way ANOVA with Duncan’s multiple range test, *p < 0.05.

Cytochrome *c* proteins were substrates for enzymatic reactions of some key enzymes, such as nitrite reductase and hydrazine dehydrogenase^22^. To further investigate the variation of cytochrome *c* under an electrostatic field, a cytochrome peroxidase stain was performed on the anammox cells collected on day 200 (intravital staining by diaminobenzidine tetrahydrochloride (DAB)). Figure 7 shows that intense staining occurs within close proximity of the anammoxosome membrane, as outlined by the dashed lines, in places where the membrane is curved. This result indicated that the cytochrome *c* proteins were predominantly located at or in close proximity to the inside of the anammoxosome membrane, which was similar to the results reported by van Niftrik *et al*.^23^. Moreover, compared to the region of the cytochrome peroxidase stain of the two images, the accumulation of an electric-dense by DAB oxidation of the anammox cells taken from R2 was much greater than R1, especially in the folds region of the anammoxosome membrane. These observations of the cytochrome *c* proteins were consistent with the measurement of the heme *c* contents. Many folds of the anammoxosome membrane amplified the superficial area and resulted in more locations for the cytochrome *c*. According to the Michaelis-Menten equation, the rate of the enzymatic reaction increases when the substrate concentration increases to a certain range. Take the HDH of anammox cells as an example, as shown in Fig. 8, in which the HDH enzymatic reaction was faster as the cytochrome *c* concentration increased. Therefore, variations of anammox cell structure caused by external electrostatic field application might favour enzymatic reaction and substrate transition and further enhanced the TN removal performance of the system.

From these results, there is some speculation about the effects of electrostatic field application on the anammox biomass. First, the electrostatic field intensity contributed to the change in the cell membrane and anammoxosome membrane, which might enlarge the membrane area to provide substrate storage. This variation of the membrane led to enhancement of substrate turnover. Second, increasing some types of substrates (e.g., cytochrome *c* proteins) of the enzymatic system accelerated several enzymatic reactions. Moreover, the acceleration of substance metabolism generated by the improvement of the catalysis of key enzymes and energy metabolism would increase the consumption of substrates such as ammonium and nitrite. Lastly, with the electrostatic field application, the relatively higher efficiency during the incubation period corresponded to long-term acceleration of the growth rate of the anammox biomass. Therefore, the effect of an electrostatic field is an important and attractive factor to consider as the universal application of the anammox process is further developed.

## Methods

### Microorganisms and feed media

The anammox biomass used for continuous experiments originated from a laboratory-scale anammox upflow column reactor in our lab. Anammox bacteria from the KSU-1 strain (AB057453.1) accounted for approximately 70–75% of the total biomass in the seed biomass based on FISH observations. The media used in the experiments mainly consisted of ammonium and nitrite in the form of (NH_4_)_2_SO_4_ and NaNO_2_. The composition of the trace mineral medium was described by van der Graaf^8^.

### Continuous experiments

**Figure 9:**
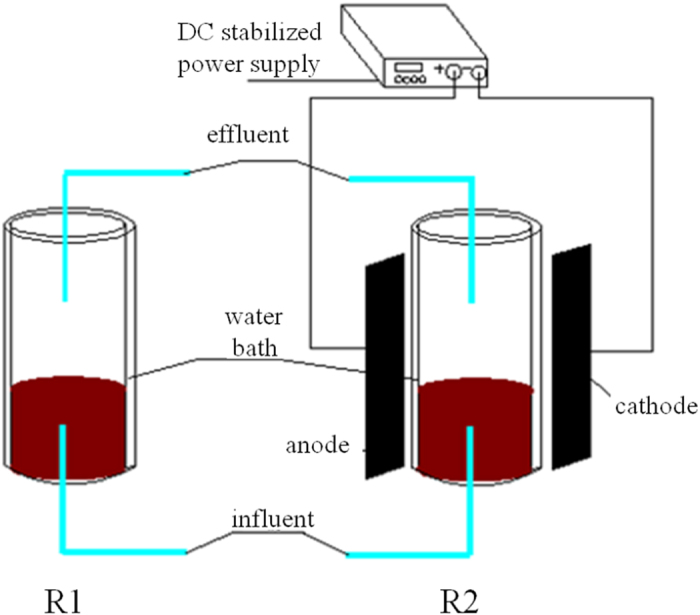
Schematic diagram of two identical anammox reactors, R2 with electrostatic field application and R1 without electrostatic field.

Two identical upflow fixed-bed column reactors, R1 (the control reactor, without an applied electrostatic field) and R2 (with an applied electrostatic field), were used for the continuous experiments. The working volumes were approximately 0.5 L with an inner diameter of 5 cm and height of 25 cm. All the reactors contained 50 g (wet weight) anammox biomass resulting in an initial MLVSS concentration of 4,920 mg/L for each reactor. Both reactors were continuously fed with the same media, and the influent was purged with 99.5% N_2_ to maintain dissolved oxygen below 0.05 mg/L. The influent pH was adjusted to 7.0 ± 0.2 by dosing with 2 M HCl and the temperature was maintained at 35 ± 1 °C using a water bath. Figure 9 shows the schematic diagram of the continuous experiments.

The application time of the external electrostatic field was 12 h per day as previously determined in this study. For operational convenience, the application time-resting time was set as 3 h-3 h, 6 h-6 h, 12 h-12 h in one cycle. The applied electrostatic field intensity of the whole continuous experiments was 2 V/cm based on preliminary experimental results. The detailed running conditions of R2 were shown in Table 2.

### Analytical methods

Concentrations of nitrite and nitrate were determined using ion-exchange chromatography (ICS-1100, Dionex, USA) with an IonPac AS18 anion column after filtration with 0.22 μm pore size membranes. NH_4_^+^-N, MLSS and MLVSS concentrations were measured according to the Standard Methods (APHA, 1995). The pH was measured using a digital pH meter (PHS-25, Leici Company, China), while DO was measured using a digital DO meter (YSI, Model 55, USA). The heme *c* content was measured according to the methods described in a reference^24^. A flow cytometry experiment (BD Biosciences, San Jose, USA) was also performed to assess the change in the cell structure and the apoptosis of the bacteria based on the methods described by Zhao *et al*.^25^. The anammox biomass samples for the transmission electron microscope (TEM) observation were taken from reactors R1 and R2 on day 200, and the observation was performed according to the methods described by Duan *et al*.^9^.

### Cytochrome peroxidase reaction

The cytochrome peroxidase reaction to observe the cytochrome *c* distribution in the anammoxosome was modified from the method of Seligman *et al*.^26^. First, the anammox cells were fixed for 30 min in 0.1 M cacodylate buffer (pH 7.2) containing 4% formaldehyde and 1.5% glutaraldehyde. Second, the cells were washed with a cacodylate buffer (pH 7.2) three times and then were incubated for 15 min with 2.5 mM diaminobenzidine tetrahydrochloride (DAB) and 0.02% H_2_O_2_ in 0.1 M cacodylate buffer (pH 6.5). Last, the cells were washed with a cold buffer containing 0.15 M sucrose (pH 7.2) to prevent the relocation of the precipitates.

### Preparation of the biomass extracts and determination of enzyme activity

At operational days 0, 17, 32, 46 and 70, 2 g (wet weight) anammox biomass was sampled from each reactor. The biomass samples were centrifuged at 8,000 rpm at 4 °C for 20 min followed by washing twice with a sodium phosphate buffer solution (20 mM, pH 7.0). The washed pellets were then resuspended in 20 ml of the same buffer and lysed by freezing and thawing, followed by sonication (225 W, at 4 °C for 30 min, Ultrasonic processor CPX 750, USA). The cell mass was separated by centrifugation (22,000 rpm), at 4 °C for 30 min. The supernatant was stored at 4 °C and used as a cell extract in the determination of the protein and enzyme activity. The protein concentration was measured according to the Bradford procedure^27^ using bovine serum albumin (BSA) as a standard. Enzyme activity of hydrazine dehydrogenase was determined according to the methods described by Shimamura *et al*.^28^, and the reactions were depicted as an increase in the absorbance of cytochrome *c* at 550 nm in the standard mixture using a spectrophotometer (V-560 UV/VIS Spectrophotometer, Jasco, Japan). The hydrazine dehydrogenase activity was expressed as μmol of cytochrome *c* reduced/mg protein/min. Nitrate reductase (Nar) activity was assayed in accordance with the methods recorded by Meincke *et al*.^29^ by measuring the consumption of nitrite. One unit of enzyme activity was defined as μmol of nitrite reduced/per protein/min. Nitrite reductase (Nir) activity was assayed on the basis of the methods described by Hira *et al*.^30^. The nitrite concentrations were detected using a spectrophotometer after 10 minutes of reaction under anaerobic conditions. A unit of enzyme activity was defined as μmol of nitrite reduced/mg protein/min.

### Quantitative PCR assay

Primer pairs Amx 809-F and Amx 1066-R (20) were used for real-time PCR quantification of the anammox bacteria. The reaction volume of 25 μL contained 12.5 μL SYBR^®^ Premix Ex Taq^TM^ (TaKaRa, Dalian, China), 0.4 mg mL-1 BSA, 200 nM final concentration of each primer, 0.5 μL Rox reference dye and 2 μL extracted DNA as a template. Three replicates were analysed for each sample. The PCR program was as follows: denaturation for 2 mins at 95 °C, followed by 40 cycles of 5 s at 95 °C, annealing for 30 s at 62 °C and elongation for 30 s at 72 °C. Melting curve analysis showed only one peak at Tm = 87.0 °C. No detectable peaks associated with primer-dimer artefacts or other nonspecific PCR amplification products were observed. The plasmid DNA concentration was determined on a Nanodrop^®^ ND-1000 UV-Vis Spectrophotometer (NanoDrop Technologies, USA). The anammox bacterial 16S rRNA gene copy number was calculated directly from the concentration of extracted plasmid DNA. Sixfold serial dilutions of a known copy number of the plasmid DNA were subjected to q-PCR assay in triplicate to generate an external standard curve.

### Statistical analysis

All the data except the influent and effluent of the reactors presented in this paper were the mean values of the data from triplicate experiments. Statistical analysis was performed using one-way ANOVA with the Duncan’s multiple range test (SPSS 19.0) and values of p < 0.05 were considered to be statistically significant.

## Additional Information

**How to cite this article**: Yin, X. *et al*. Effects of cycle duration of an external electrostatic field on anammox biomass activity. *Sci. Rep*. **6**, 19568; doi: 10.1038/srep19568 (2016).

## Figures and Tables

**Table 1:** Crude enzyme activities of both reactors during different phases.

Phase	I	II	III	IV			
Sampling day	(day15)	(day30)	(day45)	day 80	day140	day 200	day260
Crude HDH Activity (μM cytochrome *c* /min/mg protein)							
R1	1.12	1.14	1.18	1.02	1.40	1.59	1.70
R2	1.76	1.84	1.36	0.95	2.09	2.39	2.92
Crude NIR activity (μM nitrite/min/mg protein)							
R1	23.35	24.26	26.29	23.07	27.62	30.41	34.82
R2	31.89	34.52	28.10	27.38	35.34	46.53	50.78
Crude NAR activity (μM nitrite/mg protein/min)							
R1	1.70	1.80	1.98	1.80	2.10	2.75	3.26
R2	2.31	2.73	2.45	1.86	2.43	3.60	4.59

Data were shown as mean ± SD (n = 3). One-way ANOVA with Duncan’s multiple range test, *p < 0.05.

**Table 2:** The detailed running conditions of R2 during the whole continuous experiments.

Application modes	Mode 1	Mode 2	Mode 3	Mode 2
Phase	I	II	III	IV
Continuous application time—resting time	3 h-3 h	6 h-6 h	12 h-12 h	6 h-6 h
Cycles/day	4	2	1	2
Total application time per day	12 h/day			

## References

[b1] Mulder A. . Anaerobic ammonium oxidation discovered in a denitrifying fluidized bed reactor. FEMS Microbiol. Ecol. 16, 177–184 (1995).

[b2] Laureni M. . Activity and growth of anammox biomass on aerobically pre-treated municipal wastewater. Water. Res. 80, 325–336 (2015). 26024830 10.1016/j.watres.2015.04.026 PMC5250675

[b3] Op den Camp H. J. M. . Global impact and application of the anaerobic ammonium-oxidizing (anammox) bacteria. Biochem. Soc. Trans. 34, 174–178 (2006). 16417514 10.1042/BST0340174

[b4] Tang C. J. . Performance of high-loaded anammox UASB reactors containing granular sludge. Water. Res. 45, 135–144 (2010). 20801478 10.1016/j.watres.2010.08.018

[b5] Gilbert E. A. . Low temperature partial Nitritation/Anammox in a Moving bed biofilm reactor treating low strength wastewater. Environ. Sci. Technol. 48, 8784–8792 (2014). 24984033 10.1021/es501649m

[b6] Lotti T. . Faster through training: The anammox case. Water. Res. 81, 261–268 (2015). 26074189 10.1016/j.watres.2015.06.001

[b7] Strous M. . Missing lighotroph identified as new planctomycete. Nature. 400, 446–449 (1999). 10440372 10.1038/22749

[b8] Van der Star W. R. L. . Startup of reactors for anoxic ammonium oxidation: experiences from the first full-scale anammox reactor in Rotterdam. Water Res. 41, 4149–4163 (2007). 17583763 10.1016/j.watres.2007.03.044

[b9] Duan X. M. . Application of low intensity ultrasound to enhance the activity of anammox microbial consortium for nitrogen removal. Bioresour. Technol. 102, 4290–4293 (2011). 21232945 10.1016/j.biortech.2010.12.050

[b10] Liu S. T. . Enhanced anammox consortium activity for nitrogen removal: impacts of static magnetic field. J. Biotechnol. 138, 96–102 (2008). 18775754 10.1016/j.jbiotec.2008.08.002

[b11] Chang D. C. Cell poration and cell fusion using an oscillating electrostatic field. Biophysical J. 56, 641–652 (1989). 10.1016/S0006-3495(89)82711-0 PMC1280520 2819230

[b12] Funk R. H. W. & Monsees T. K. Effects of electromagnetic fields on cells: physiological and therapeutical approaches and molecular mechanisms of interaction. Cells Tissues Organs. 182, 59–78 (2006). 16804297 10.1159/000093061

[b13] Amarjargal A. . Inactivation of bacteria in batch suspension by fluidized ceramic tourmaline nanoparticles under oscillating radio frequency electrostatic fields. Ceram. Int. 39, 2141–2145 (2013).

[b14] Wouters P. C. . Critical factors determining inactivation kinetics by pulsed electrostatic field food processing. Trends Food Sci. Technol. 12, 112–121 (2001).

[b15] Meller R. B. & Campbell W. H. Reduction of nitrate and nitrite in water by immobilized enzymes. Nature. 355, 717–719 (1992).

[b16] Qiao S. . Inhibition and recovery of continuous electrostatic field application on the activity of anammox biomass. Biodegradation 25, 505–513 (2014). 24258098 10.1007/s10532-013-9677-7

[b17] Strous M. . The sequencing batch reactor as a powerful tool for the study of slowly growing anaerobic ammonium-oxidizing microorganisms. Appl. Microbiol. Biotechnol. 50, 589–596 (1998).

[b18] Neumann S. . Isolation and characterization of a prokaryotic cell organelle from the anammox bacterium Kuenenia stuttgartiensis. Mol. Microbiol. 94, 794–802 (2014). 25287816 10.1111/mmi.12816

[b19] Jetten M. S. M. . Biochemistry and cular biology of anammox bacteria. Crit. Rev. Biochem. Mol. Biol. 44, 65–84 (2009). 19247843 10.1080/10409230902722783

[b20] Neumann S. . The ultrastructure of the compartmentalizedanaerobic ammonium-oxidizing bacteria is linked to their energy metabolism. Biochem. Soc. Trans. 39, 1805–1810 (2011). 22103530 10.1042/BST20110728

[b21] Bowman S. E. J. & Bren K. L. The chemistry and biochemistry of Heme *c*: functional bases for covalent attachment. Nat. Prod. Rep. 25, 1118–1130 (2008). 19030605 10.1039/b717196j PMC2654777

[b22] Kartal B. . Molecular mechanism of anaerobic ammonium oxidation. Nature 479, 127–130 (2011). 21964329 10.1038/nature10453

[b23] Van Niftrik L. A. . Linking ultrastructure and function in four genera of anaerobic ammonium-oxidizing bacteria: cell plan, glycogen storage, and localization of cytochrome *c* proteins. J. Biotechnol. 190, 708–717 (2008). 10.1128/JB.01449-07 PMC2223682 17993524

[b24] Berry E. A. & Trumpower B. L. Simultaneous determination of hemes a, b, and c from pyridine hemochrome spectra. Anal. Biochem. 161, 1–15 (1987). 3578775 10.1016/0003-2697(87)90643-9

[b25] Zhao W. . Quantitative and real time detection of pulsed electrostatic field induced damage on Escherichia coli cells and sublethally injured microbial cells using flow cytometryin combination with fluorescent techniques. Food C. 22, 566–573 (2011).

[b26] Seligman A. M. . Nondroplet ultrastructural demonstration of cytochrome oxidase activity with a polymerizing osmiophilic reagent, diaminobenzidine (DAB). J. Cell Biol. 38, 1–14 (1968). 4300067 10.1083/jcb.38.1.1 PMC2107464

[b27] Bradford M. M. A rapid and sensitive for the quantitation of microgram quantitites of protein utilizing the principle of protein-dye binding. Anal. Biochem. 72, 248–254 (1976). 942051 10.1016/0003-2697(76)90527-3

[b28] Shimamura M. . Isolation of a multiheme protein with features of a hydrazine-oxidizing enzyme from an anaerobic ammonium-oxidizing enrichment culture. Appl. Environ. Microbiol. 73, 1065–1072 (2007). 17172456 10.1128/AEM.01978-06 PMC1828659

[b29] Meincke M. . Nitrite oxidoreductase from Nitrobacter hamburgensis: redox centers and their catalytic role. Arch. Microbiol. 158, 127–131 (1992).

[b30] Hira D. . Anammox organism KSU-1 expresses a NirK-type copper-containing nitrite reductase instead of a NirS-type with cytochrome *cd1*. FEBS Lett. 586, 1658–1663 (2012). 22673575 10.1016/j.febslet.2012.04.041

